# Supplementary material for: Development of a DNA Metabarcoding Method for the Identification of Insects in Food
Source: Foods. 2023 Mar 3;12(5):1086. doi: 10.3390/foods12051086 (PMC10001320; doi:10.3390/foods12051086)
Supplement: Supplementary file 1 [file foods-12-01086-s001.zip › Supplementary Table S1_database entries Insect DNA sequences.pdf]

foods

MDPI

Appendix A

Supplementary Table S1: List of insect DNA sequences (mitochondrial 16S rDNA) downloaded from NCBI for AGES customized database (accession numbers and latin name)

NC_026132 Formica fusca
NC_042676 Camponotus concavus
NC_029357 Camponotus atrox
NC_026711 Formica selysi
MF_417380 Atta texana
KC_346251 Atta laevigata
MT_862424 Lasius niger
BK_012808 Acromyrmex octospinosus
NC_049859 Aphaenogaster famelica
NC_026133 Myrmica scabrinodis
NC_014672 Solenopsis invicta
NC_014669 Solenopsis geminata
NC_046399 Acropyga smithii
NC_014677 Solenopsis richteri
NC_046421 Acropyga fuhrmanni
NC_041075 Dolichoderus sibiricus
NC_023093 Leptomyrmex pallens
NC_046420 Acropyga goeldii
NC_030790 Polyrhachis dives
NC_045057 Linepithema humile
NC_051486 Monomorium pharaonis
NC_046422 Acropyga guianensis
NC_015075 Pristomyrmex punctatus
NC_030176 Vollenhovia emeryi
NC_030541 Wasmannia auropunctata
NC_046426 Acropyga panamensis
NC_046424 Acropyga myops
NC_046425 Acropyga pallida
NC_039576 Anoplolepsis gracilipes
NC_046398 Acropyga sauteri
NC_046423 Acropyga kinomurai
NC_053900 Colobopsis nipponica
AF_466146 Melipona bicolor
AP_018403 Apis mellifera carpatica
NC_035883 Apis mellifera sahariensis
AP_018432 Apis mellifera buckfast
NC_034003 Vespa orientalis
CM_022150 Frieseomelitta varia

NC_050197 Vespa mandarinia 43
NC_046020 Vespa simillima simillima 44
NC_045283 Bombus waltoni 45
NC_045215 Vespula flaviceps 46
FR_997693 Vespula vulgaris 47
NC_045179 Bombus terrestris terrestris 48
NC_045178 Bombus terrestris lusitanicus 49
NC_039709 Apis andreniformis 50
NC_039134 Vespa affinis 51
NC_038114 Apis nigrocincta 52
NC_037709 Apis dorsata 53
NC_036235 Apis nuluensis 54
NC_036155 Apis laboriosa 55
NC_035146 Vespa velutina 56
NC_026198 Melipona scutellaris 57
MT_645078 Nomia chalybeata 58
MT_188686 Apis mellifera anatoliaca 59
MT_137096 Vespa velutina auraria 60
NC_028017 Megachile sculpturalis 61
NC_021401 Apis florea 62
NC_014295 Apis cerana 63
NC_011923 Bombus hypocrita 64
NC_010967 Bombus ignitus 65
NC_001566 Apis mellifera ligustica 66
MN_733955 Apis mellifera sinisxinyuan 67
MN_714162 Apis mellifera ruttneri 68
MN_714161 Apis mellifera jemenitica 69
MN_714160 Apis mellifera caucasica 70
MN_585110 Apis mellifera iberiensis 71
MN_585109 Apis mellifera adansonii 72
MN_585108 Apis mellifera simensis 73
MN_119925 Apis mellifera unicolor 74
MK_994526 Neodiprion sertifer 75
MK_440075 Vespa basalis 76
LC_519884 Polistes riparius 77
MF_678581 Apis mellifera monticola 78
MF_995069 Bombus consobrinus 79
KY_464958 Apis mellifera lamarckii 80
KY_464957 Apis mellifera meda 81
KY_091645 Vespa velutina nigrithorax 82
KX_950825 Vespa ducalis 83
KX_870183 Apis mellifera capensis 84

KP_163643 Apis mellifera syriaca	85
KM_458618 Apis mellifera intermissa	86
KJ_601784 Apis mellifera scutellata	87
AP_018434 Apis mellifera mellifera	88
MN_250878 Apis mellifera carnica	89
NC_017748 Extatosoma tiaratum	90
NC_048984 Hierodula membranacea	91
NC_034283 Hierodula patellifera	92
NC_030266 Tenodera sinensis	93
NC_030265 Mantis religiosa	94
NC_030264 Humbertiella nada	95
NC_029326 Hierodula formosana	96
NC_014688 Megacrania alpheus adan	97
NC_014694 Entoria okinawaensis	98
NC_014705 Phraortes sp.	99
NC_014702 Ramulus irregulariterdentatus	100
NC_014680 Heteropteryx diatata	101
NC_014673 Micadina phluctainoides	102
NC_014695 Phraortes illepidus	103
NC_014678 Phobaeticus serratipes	104
NC_013185 Ramulus hainanense	105
NC_056843 Mesopteryx alata	106
NC_030268 Anaxarcha zhengi	107
NC_034282 Rhombodera brachynota	108
NC_056850 Phyllothelys werneri	109
NC_034284 Rhombodera valida	110
NC_056856 Hierodula multispina	111
NC_056859 Pseudovates chlorophaea	112
NC_051892 Arria pallida	113
NC_056877 Acromantis hesione	114
NC_007702 Tamolanica tamolana	115
NC_056865 Theopompa milligratulata	116
NC_037207 Schizocephala bricornis	117
NC_056872 Hestiasula hoffmanni	118
NC_056849 Hierodula majuscula	119
NC_037697 Paratoxodera polyacantha	120
NC_056867 Pnigomantis medioconstricta	121
NC_056873 Sinomiopteryx grahami	122
NC_051490 Pliacanthopus bimaculatus	123
NC_051489 Rhombodera longa	124
NC_056840 Statilia maculata	125
NC_056857 Sinomantis denticulata	126

NC_037235 Sibylla pretiosa	127
NC_056864 Amorphoscelis chinensis	128
NC_037234 Creobroter jiangxiensis	129
NC_057070 Amorphoscelis hainana	130
NC_056846 Spilomantis occipitalis	131
NC_056839 Miomantis caffra	132
NC_056862 Orthodera ministralis	133
NC_030267 Creobroter gemmatus	134
NC_056860 Popa spurca	135
NC_056869 Phyllocrania paradoxa	136
NC_037203 Amantis nawai	137
NC_056855 Theopropus cattulus	138
NC_056866 Deroplatys lobata	139
NC_056874 Hymenopus coronatus	140
NC_056878 Anarxarcha sinensis	141
NC_056863 Acontista multicolor	142
NC_056854 Blepharopsis mendica	143
NC_037381 Toxodera hauseri	144
NC_037205 Tropidomantis tenera	145
NC_056851 Idlomorpha lateralis	146
NC_056879 Creobroter apicalis	147
NC_056880 Parymenopus davisoni	148
NC_056848 Theopropus rubrobrunneus	149
NC_056870 Prohierodula picta	150
NC_037206 Sceptuchus simplex	151
NC_056841 Miromantis yunnanensis	152
NC_024028 Leptomantella albella	153
NC_056868 Tenodera angustipennis	154
NC_056861 Sphodromantis viridis	155
NC_056881 Pseudempusa pinnapavonis	156
NC_045876 Psychomantis borneensis	157
NC_056844 Parasphendale agrionina	158
NC_056852 Empusa pennicornis	159
NC_056847 Taumantis sigiana	160
NC_037204 Sphodromantis lineola	161
NC_037208 Eomantis yunnanensis	162
NC_056875 Tarachomantis alaotrana	163
NC_056871 Neodanuria bolauana	164
NC_056853 Brunneria borealis	165
NC_056845 Gongylus gongylodes	166
NC_056858 Deroplatys desiccata	167
NC_056842 Pseudocreobotra wahlbergii	168

NC_056876 Idolomantis diabolica	169
DQ_241796 Grylloblatta sculleni	170
NC_007701 Sclerophasma paresisense	171
DQ_241799 Timema californicum	172
NC_057442 Tachycines zorzini	173
NC_057646 Melanoplus differentialis	174
NC_056785 Chorthippus parallelus parallelus	175
NC_056786 Chorthippus parallelus erythropus	176
NC_057053 Gryllus veletis	177
NC_057052 Gryllus lineaticeps	178
NC_057195 Gryllodes sigillatus	179
NC_056238 Sinopodisma qinlingensis	180
NC_053745 Oxytauchira flange	181
NC_053658 Fer nigripennis	182
NC_053659 Caryandoides hunanica	183
NC_053660 Paratoacris reticulipennis	184
NC_053383 Euconocephalus nasutus	185
NC_052715 Anapodisma miramae	186
NC_052716 Sinopodisma rostellocerca	187
NC_052717 Stenocatantops mistshenkoi	188
NC_052731 Bryodema kozlovi	189
NC_052732 Epacromius coerulipes	190
NC_052733 Filchnerella rubrimargina	191
NC_052734 Oedaleus manjius	192
NC_051867 Sinopodisma pieli	193
NC_050742 Natula pravdini	194
NC_048465 Chorthippus fallax	195
NC_048466 Shoveliteratura triangula	196
NC_046894 Anterastes babadaghi	197
NC_046411 Gesonula punctifrons	198
NC_046412 Tetrix ruyuanensis	199
NC_046527 Apalacris nigrogeniculata	200
NC_046528 Conophymacris viridis	201
NC_046529 Indopodisma kingdoni	202
NC_046530 Paratonkinacris vittifemoralis	203
NC_046532 Spathosternum prasiniferum prasiniferum	204
NC_046533 Xiangelilacris zhongdianensis	205
NC_046534 Pseudoeoscyllina brevipennisoides	206
NC_046535 Bryodema nigroptera	207
NC_046536 Bryodemella holdereri holdereri	208
NC_046537 Heteropternis respondens	209
NC_046539 Parapleurus alliaceus	210

NC_046540 Ergatettix dorsifera	211
NC_046543 Bryodema dolichoptera	212
NC_046545 Fruhstorferiola omei	213
NC_046546 Sinopodisma funiushana	214
NC_046547 Sinopodisma wudangshanensis	215
NC_046548 Ruidocollaris convexipennis	216
NC_046549 Sinopodisma lushiensis	217
NC_046550 Sphingonotus menglaensis	218
NC_046553 Bryodemacris uvarovi	219
NC_046554 Bryodemella tuberculata diluta	220
NC_046555 Dericorys annulata	221
NC_046556 Emeiacris maculata	222
NC_046557 Euthystira luteifemora	223
NC_046558 Filchnerella qilianshanensis	224
NC_046559 Filchnerella tenggerensis	225
NC_046560 Omocestus viridulus	226
NC_046561 Pedopodisma emeiensis	227
NC_046562 Sinopodisma lofaoshana	228
NC_046563 Sphingonotus ningsianus	229
NC_046564 Sphingonotus yenchihensis	230
NC_046565 Traulia orchotibialis	231
NC_046570 Oxytauchira brachyptera	232
NC_045846 Dianemobius fascipes	233
NC_045847 Dianemobius furumagiensis	234
NC_045848 Polionemobius taprobanensis	235
NC_045930 Tagasta indica	236
NC_045237 Euchorthippus unicolor	237
NC_045212 Acosmetura nigrogeniculata	238
NC_042665 Poecilimon luschani	239
NC_042666 Isophya major	240
NC_041412 Dasyhippus barbipes	241
NC_041236 Xenogryllus marmoratus	242
NC_041114 Traulia nigritibialis	243
NC_041116 Choroedocus capensis	244
NC_040974 Xizicus maculatus	245
NC_039981 Xiphidiopsis gurneyi	246
NC_039664 Cacoplistes rogenhoferi	247
NC_039665 Meloimorpha japonica	248
NC_039666 Ornebius bimaculatus	249
NC_039667 Ornebius kanetataki	250
NC_039739 Ornebius fuscicerci	251
NC_039408 Dnopherula yuanmowensis	252

NC_037914 Cardiodactylus muiri 253
NC_036994 Longchuanacris curvifurculus 254
NC_036750 Caryanda elegans 255
NC_036063 Taulia minuta 256
NC_035552 Pteranabropsis carnarius 257
NC_035553 Pteranabropsis crenatis 258
NC_035227 Pternoscirta caliginosa 259
NC_035420 Pteranabropsis carli 260
NC_034994 Sinochlora szechwanensis 261
NC_034673 Choroedocus violaceipes 262
NC_034756 Phaneroptera gracilis 263
NC_034757 Phaneroptera nigroantennata 264
NC_034797 Truljalia hibinonis 265
NC_034799 Oecanthus sinensis 266
NC_033853 Pseudocosmetura anjiensis 267
NC_033905 Sinopodisma houshana 268
NC_033906 Sinopodisma wulingshanensis 269
NC_033981 Decma fissa 270
NC_033982 Pseudokuzicus pieli 271
NC_033984 Zichya baranovi 272
NC_033986 Metrioptera bonneti 273
NC_033987 Conanalus pieli 274
NC_033990 Pseudorhynchus crassiceps 275
NC_033991 Ruspolia lineosa 276
NC_033992 Pseudorhynchus acuminatus 277
NC_033994 Phyrganogryllacris xiai 278
NC_033995 Kuwayamaea chinensis 279
NC_033996 Lipotactes tripyrga 280
NC_033997 Phyllomimus sinicus 281
NC_033998 Homogryllacris anelytra 282
NC_032716 Tonkinacris sinensis 283
NC_032303 Sinopodisma tsinlingensis 284
NC_032077 Trigonidium sjostedti 285
NC_031817 Fruhstorferiola tonkinensis 286
NC_031379 Fruhstorferiola huayinensis 287
NC_031397 Curvipennis wixiensis 288
NC_030586 Yunnanacris yunnaneus 289
NC_030165 Caryanda sp. 290
NC_029408 Compsorhipis davidiana 291
NC_029135 Peripolus nepalensis 292
NC_029205 Gonista bicolor 293
NC_028158 Phyllomimus detersus 294

NC_028160 Ruidocollaris obscura 295
NC_028059 Cyphoderris monstrosa 296
NC_028060 Camptonotus carolinensis 297
NC_028061 Pseudothericles compressifrons 298
NC_028062 Comicus campestris 299
NC_028065 Mirhipipteryx andensis 300
NC_027187 Qinlingacris taibaiensis 301
NC_026716 Fruhstorferiola kulinga 302
NC_026525 Sinotmethis brachypterus 303
NC_025946 Angaracris rhodopa 304
NC_025765 Pseudoxya diminuta 305
NC_025558 Angaracris barabensis 306
NC_024923 Filchnerella beicki 307
NC_023920 Kingdonella bicollina 308
NC_023921 Mekongiella kingdoni 309
NC_023919 Pacris xizangensis 310
NC_023535 Humphaplotropis culaishanensis 311
NC_023467 Orinhippus tibetanus 312
NC_021609 Xenocatantops brachycerus 313
NC_021397 Tarragoilus diuturnus 314
NC_021424 Sinochlora longifissa 315
NC_021103 Gomphocerus sibiricus 316
NC_020773 Tristira magellanica 317
NC_020774 Lentula callani 318
NC_020775 Lithidiopsis carinatus 319
NC_020776 Pyrgacris descampsi 320
NC_020777 Tanaocerus koebelei 321
NC_020778 Ommexecha virens 322
NC_020328 Asiotmethis zacharjini 323
NC_020329 Filchnerella helanshanensis 324
NC_020330 Pseudotmethis rubimarginis 325
NC_019993 Chondracris rosea 326
NC_018765 Xizicus fascipes 327
NC_018542 Alulatettix yunnanensis 328
NC_018543 Tetrix japonica 329
NC_016182 Pielonastax zhengi 330
NC_015478 Gomphocerus sibiricus tibetanus 331
NC_014610 Thrinchus schrenkii 332
NC_014488 Ellipes minuta 333
NC_014491 Physemacris variolosa 334
NC_014449 Euchorthippus fusigeniculatus 335
NC_014450 Mekongiana xiangchengensis 336

NC_014451 Mekongiella xizangensis 337
NC_014349 Gomphocerippus rufus 338
NC_013805 Arcyptera coreana 339
NC_013826 Traulia szetschuanensis 340
NC_013835 Prumna arctica 341
NC_013847 Gomphocerus licenti 342
NC_013701 Ognevia longipennis 343
NC_011813 Deracantha onos 344
NC_011306 Troglophilus neglectus 345
NC_011115 Oedaleus asiaticus 346
NC_011095 Megaulacobothrus chinensis 347
NC_006678 Gryllotalpa orientalis 348
NC_053942 Uvaroviola multispinosa 349
CM_015047 Drosophila innubila 350
NC_039437 Thrips palmi 351
KP_861632 Chrysomya pacifica 352
KU_588141 Drosophila suzukii 353
MK_290826 Nemoura papilla 354
MK_893470 Calliphora nigribarbis 355
MN_400755 Kamimuria klapaleki 356
MN_551230 Drosophila antonietae 357
MN_551231 Drosophila borborema 358
MN_551232 Drosophila buzzatii 359
MN_551235 Drosophila seriema 360
MN_912823 Eristalis cerealis 361
MT_677866 Stenopsyche angustata 362
NC_000857 Ceratitis capitata 363
NC_001322 Drosophila yakuba 364
NC_002697 Chrysomya putoria 365
NC_005333 Bactrocera oleae 366
NC_005779 Drosophila mauritiana 367
NC_005780 Drosophila sechellia 368
NC_005781 Drosophila simulans 369
NC_042712 Bactrocera biguttula 370
NC_050320 Simulium noelleri 371
NC_050319 Tipula fascipennis 372
NC_046952 Bactrocera ruiliensis 373
NC_046603 Drosophila pseudoobscura 374
NC_038167 Isoperla eximia 375
NC_046521 Bactrocera rubigina 376
NC_044669 Drosophila mercatorum 377
NC_041143 Eristalis tenax 378

NC_011276 Corydalus cornutus 379
NC_040120 Simulium maculatum 380
NC_038190 Isoperla bilineata 381
NC_038164 Bactrocera tsuneonis 382
NC_034912 Anastrepha fraterculus 383
NC_011524 Protohermes concolorus 384
NC_029466 Bactrocera latifrons 385
NC_028226 Delia antiqua 386
NC_024855 Musca domestica 387
NC_024511 Drosophila melanogaster 388
NC_009772 Bactrocera carambolae 389
NC_006133 Pteronarcys princeps 390
NC_037910 Musca sorbens 391
NC_037723 Bactrocera ritsemai 392
NC_037722 Bactrocera limbifera 393
NC_035232 Hermetia illucens 394
NC_034939 Nemoura nankinensis 395
NC_033348 Simulium variegatum 396
NC_031381 Chrysomya phaonis 397
NC_030520 Tipula cockerelliana 398
NC_029753 Simulium aureohirtum 399
NC_029468 Bactrocera umbrosa 400
NC_008748 Bactrocera dorsalis 401
NC_029467 Bactrocera melastomatos 402
NC_029215 Calliphora chinghaiensis 403
NC_028518 Drosophila formosana 404
NC_028412 Chrysomya nigripes 405
NC_028411 Calliphora vomitoria 406
NC_028347 Bactrocera diaphora 407
NC_028327 Bactrocera arecae 408
NC_028076 Kamimuria chungnanshana 409
NC_027937 Drosophila albomicans 410
NC_027725 Bactrocera zonata 411
NC_025936 Drosophila incompta 412
NC_025338 Chrysomya pinguis 413
NC_023825 Drosophila santomea 414
NC_023462 Acanthacorydalis orientalis 415
NC_019639 Calliphora vicina 416
NC_019635 Chrysomya saffranea 417
NC_019634 Chrysomya rufifacies 418
NC_019633 Chrysomya megacephala 419
NC_019632 Chrysomya bezziana 420

NC_019631 Chrysomya albiceps 421
NC_018787 Bactrocera correcta 422
NC_016056 Bactrocera cucurbitae 423
NC_014611 Bactrocera tryoni 424
NC_014402 Bactrocera minax 425
NC_013932 Hypoderma lineatum 426
NC_012645 Ephemera orientalis 427
NC_011596 Drosophila littoralis 428
NC_009733 Lucilia sericata 429
NC_028057 Lucilia Caesar 430
NC_012644 Davidius lunatus 431
NC_057490 Epeorus dayongensis 432
NC_039612 Epeorus herklotsi 433
NC_050279 Ephemerella sp. Yunnan 434
NC_042163 Isonychia kiangsinensis 435
NC_011359 Parafronurus youi 436
NC_050280 Serratella sp. Liaoning 437
NC_050281 Serratella sp. Yunnan 438
NC_050282 Serratella zapekinae 439
NC_013822 Siphlonurus immanis 440
NC_050283 Torleya grandiforceps 441
NC_050284 Torleya nepalica 442
NC_053852 Acroneuria carolinensis 443
NC_026104 Acroneuria hainana 444
NC_057056 Amphinemura bulla 445
NC_044748 Amphinemura longispina 446
NC_044749 Amphinemura yao 447
NC_042199 Antarctoperla michaelseni 448
NC_027698 Apteroperla tikumana 449
NC_034661 Capnia zijinshana 450
NC_042205 Diamphipnoa annulata 451
NC_022843 Dinocras cephalotes 452
NC_057436 Flavoperla hatakeyamae 453
NC_044750 Indonemoura jacobsoni 454
NC_044751 Indonemoura nohirae 455
NC_044719 Mesonemoura metafiligera 456
NC_057512 Sphaeronemoura elephas 457
NC_057513 Nemoura meniscata 458
NC_057280 Paragnetina indentata 459
NC_057281 Perlesta teaysia 460
NC_053853 Togoperla limbata 461
NC_053557 Paraleuctra cercia 462

NC_053558 Perlomyia isobeae	463
NC_050322 Protonemura meyeri	464
NC_044720 Mesonemoura tritaenia	465
NC_044752 Protonemura kohnoae	466
NC_044753 Protonemura orbiculata	467
NC_044754 Sphaeronemoura grandicauda	468
NC_044755 Sphaeronemoura hamistyla	469
NC_042200 Neuroperla schedingi	470
NC_042206 Neonemura barrosi	471
NC_042207 Rhopalopsole bulbifera	472
NC_038168 Pseudomegarcys japonica	473
NC_038189 Soliperla sp.	474
NC_037754 Suwallia teleckojensis	475
NC_037897 Taeniopteryx ugola	476
NC_034997 Zelandoperla fenestrata	477
NC_034809 Styloperla spinicercia	478
NC_029248 Pteronarcella badia	479
NC_056285 Oyamia nigribasis	480
NC_044742 Panorpa debilis	481
NC_015118 Bittacus pilicornis	482
NC_044741 Bittacus strigosus	483
NC_013180 Neopanorpa pulchra	484
NC_029246 Hydropsyche pellucidula	485
NC_026219 Limnephilus decipiens	486
NC_050311 Potamophylax latipennis	487
NC_016173 Paracladura trichoptera	488
NC_058007 Stenopsyche tienmushanensis	489
NC_051530 Macrostemum floridum	490
NC_036156 Hydromanicus wulaianus	491
NC_036950 Hydropsyche simulans	492
NC_036951 Hydropsyche orris	493
NC_044710 Limnephilus hyalinus	494
NC_043771 Phryganopsyche latipennis	495
NC_036953 Potamyia flava	496
NC_036952 Cheumatopsyche speciosa	497
NC_036954 Cheumatopsyche campyla	498
NC_036955 Cheumatopsyche analis	499
NC_036004 Anabolia bimaculata	500
NC_043770 Hydatophylax nigrovittatus	501
NC_039714 Phryganea cinerea	502
NC_023374 Eubasilissa regina	503
NC_039659 Triaenodes tardus	504

NC_050321 Phryganea bipunctata 505
NC_013251 Mongoloraphidia harmandi 506
NC_053680 Sarcophaga cetu 507
NC_053665 Sarcophaga carnaria 508
MK_659821 Drosophila hydei 509
NC_019636 Protophormia terraenovae 510
LC_521855 Teleogryllus occipitalis 511
NC_053546 Gryllus bimaculatus 512
MZ_440654 Acheta domesticus 513
MF_347703 Conocephalus differentus 514
NC_046552 Atractomorpha psittacina 515
NC_046544 Calliptamus barbarus 516
NC_046542 Euparatettix variabilis 517
NC_046541 Euparatettix bimaculatus 518
NC_046538 Oedaleus abruptus 519
NC_046531 Shirakiacris yunkweiensis 520
NC_045928 Oxya hainanensis 521
NC_045883 Oxya agavisa 522
NC_045841 Homoeoxipha nigripes 523
NC_045065 Conocephalus maculatus 524
NC_043956 Ceracris fasciata fasciata 525
NC_043773 Oxya japonica 526
NC_042904 Diabolocatantops pinguis 527
NC_033993 Holochlora fruhstorferi 528
NC_041115 Stenocatantops splendens 529
NC_036062 Nomadacris japonica 530
NC_034773 Pseudophyllus titan 531
NC_034674 Aiolopus thalassinus 532
NC_033999 Hexacentrus unicolor 533
NC_033989 Diestrammena asynamora 534
NC_033988 Conocephalus melaenus 535
MK_903561 Gampsocleis sedakovii 536
NC_033985 Loxoblemmus doenitzi 537
NC_033983 Hexacentrus japonicus 538
NC_032076 Oxya hyla 539
NC_001712 Locusta migratoria 540
NC_014891 Locusta migratoria manilensis 541
MK_903597 Phlaeoba antennata 542
NC_031652 Ducetia japonica 543
NC_031506 Phlaeoba infumata 544
MG_993444 Cyrtacanthacris tatarica 545
NC_030763 Loxoblemmus equestris 546

NC_030762 Velarifictorus hemelytrus 547
NC_030626 Calliptamus abbreviatus 548
NC_030587 Hieroglyphus tonkinensis 549
NC_029327 Oedaleus infernalis 550
NC_029150 Phlaeoba tenebrosa 551
NC_009967 Anabrus simplex 552
NC_029148 Gryllotalpa unispina 553
NC_028619 Teleogryllus oceanicus 554
NC_028063 Henicus brevimucronatus 555
NC_028058 Stenopelmatus fuscus 556
NC_011114 Gastrimargus marmoratus 557
NC_027179 Trilophidia annulata 558
NC_025285 Ceracris versicolor 559
NC_021610 Shirakiacris shirakii 560
NC_021380 Mecopoda elongata 561
NC_021379 Mecopoda niponensis 562
NC_019994 Ceracris kiangsu 563
NC_015624 Locusta migratoria tibetensis 564
NC_014887 Acrida cinerea 565
NC_009876 Ruspolia dubia 566
NC_014490 Xyleus modestus 567
NC_013240 Schistocerca gregaria gregaria 568
NC_011827 Phlaeoba albonema 569
NC_011824 Atractomorpha sinensis 570
NC_011823 Teleogryllus emma 571
NC_011305 Calliptamus italicus 572
NC_011303 Acrida willemsei 573
NC_010219 Oxya chinensis 574
NC_011302 Gryllotalpa pluvialis 575
NC_011200 Gampsocleis gratiosa 576
NC_011119 Locusta migratoria migratoria 577
NC_011301 Myrmecophilus manni 578
MK_446423 Gryllus alexanderi 579
NC_003081 Tribolium castaneum 580
MN_580549 Lucanus cervus 581
NC_048951 Neoplocaederus obesus 582
KT_070712 Cryptolestes turcicus 583
KX_087289 Epuraea guttata 584
NC_049092 Alphitobius diaperinus 585
MN_829437 Dorysthenes granulosus 586
NC_044849 Dermestes tessellatocollis 587
KF_658070 Diabrotica virgifera 588

KF_364622 Prosopocoilus astacoides blanchardi 589
KT_876878 Acilius sulcatus 590
KT_876883 Berosus affinis 591
NC_044757 Dytiscus sharpi 592
MK_134566 Prosopocoilus bulbosus mandibularis 593
MT_457815 Oryctes rhinoceros 594
MK_134565 Prosopocoilus laterotarsus maedaorum 595
MT_371041 Aromia bungii 596
MF_908524 Odontolabis cuvera 597
KT_876890 Haliplus lineatocollis 598
MT_880604 Mylabris calida 599
KT_876889 Haliplus immaculatus 600
MK_614550 Agrianome spinicollis 601
MK_250907 Lucanus maculifemoratus taiwanus 602
MT_809476 Sipalinus gigas 603
NC_045101 Ceutorhynchus assimilis 604
KT_876887 Dorcus parallelipipedus 605
NC_022935 Diabrotica barberi 606
NC_013070 Psacothea hilaris 607
NC_028203 Cryptolestes ferrugineus 608
NC_013578 Lucanus mazama 609
NC_022671 Batocera lineolata 610
NC_045123 Dorcus ursulus 611
NC_045124 Dorcus tenuihirsutus 612
NC_045923 Copris tripartitus 613
NC_047449 Blaps rhynchoptera 614
NC_044713 Anthonomus rectirostris 615
NC_044714 Anthonomus rubi 616
NC_045097 Xystrocera globosa 617
NC_045068 Cyclommatus strigiceps 618
NC_044961 Lucanus fortunei 619
NC_044851 Dermestes coarctatus 620
NC_044850 Dermestes frischii 621
NC_038089 Aegosoma sinicum 622
NC_037927 Dorysthenes paradoxus 623
NC_042151 Aristobia reticulator 624
NC_043883 Glenea cantor 625
NC_044711 Anthonomus eugenii 626
NC_044712 Anthonomus pomorum 627
NC_037200 Dermestes maculatus 628
NC_037698 Callipogon relictus 629
NC_041101 Zophobas atratus 630

NC_039705 Cyrtotrachelus buqueti 631
NC_038191 Cicindela anchoralis 632
NC_038115 Popillia japonica 633
NC_028535 Rhynchophorus ferrugineus 634
NC_028204 Cryptolestes pusillus 635
NC_035677 Acanthoscelides obtectus 636
NC_036038 Prosopocoilus confucius 637
NC_036046 Mylabris aulica 638
NC_037196 Tenebrio obscurus 639
NC_027580 Prosopocoilus gracilis 640
NC_033872 Apriona swainsoni 641
NC_030782 Xylotrechus grayii 642
NC_030765 Sitophilus oryzae 643
NC_030764 Sitophilus zeamais 644
NC_029230 Anoplophora chinensis 645
NC_012765 Chrysochroa fulgidissima 646
NC_009964 Pyrophorus divergens 647
NC_008221 Anoplophora glabripennis 648
NC_023453 Protaetia brevitarsis 649
NC_024600 Tribolium audax 650
NC_024633 Tenebrio molitor 651
NC_025332 Ulomoides dermestoides 652
MK_878514 Lucanus chengyuani 653
NC_057491 Epeorus carinatus 654
MF_191873 Xylotrupes siamensis 655
JX_234466 Pachnoda marginata marginata 656
MF_716899 Enallagma cyathigerum 657
MT_408029 Anax parthenope 658
NC_042691 Tramea virginia 659
NC_042732 Orthetrum melania 660
NC_046756 Epophthalmia elegans 661
NC_026305 Brachythemis contaminata 662
NC_031821 Anax imperator 663
NC_032047 Orthetrum glaucum 664
NC_032048 Orthetrum chrysis 665
NC_032049 Orthetrum sabina 666
NC_032050 Orthetrum testaceum 667
NC_042689 Acisoma panorpoides 668
NC_027181 Atrocalopteryx atrata 669
NC_036749 Atrocalopteryx melli 670
NC_054209 Ceriagrion fallax 671
NC_042690 Deielia phaon 672

NC_023232 Epiophlebia superstes 673
NC_026058 Euphaea decorata 674
NC_014493 Euphaea formosa 675
NC_026059 Euphaea ornata 676
NC_026057 Euphaea yayeyamana 677
NC_025758 Hydrobasileus croceus 678
NC_031824 Ischnura elegans 679
NC_021617 Ischnura pumilio 680
NC_050696 Libellula angelina 681
NC_041425 Macromia daimoji 682
NC_031823 Megaloprepus caerulatus 683
NC_057643 Mnais tenuis 684
NC_039410 Nannophya pygmaea 685
NC_053718 Neurothemis fulvia 686
NC_056355 Pantala flavescens 687
NC_027180 Platycnemis foliacea 688
NC_020636 Pseudolestes mirabilis 689
NC_053716 Pseudothemis zonata 690
NC_042442 Psolodesmus mandarinus 691
NC_042733 Tridomphus carus 692
NC_023233 Vestalis melania 693
NC_034842 Neostylopyga rhombifolia 694
NC_012901 Blattella germanica 695
NC_006076 Periplaneta fuliginosa 696
NC_016956 Periplaneta americana 697
NC_037496 Cryptocercus meridianus 698
NC_039940 Periplaneta brunnea 699
NC_018132 Cryptocercus relictus 700
NC_018549 Blattella bisignata 701
NC_030001 Gromphadorhina portentosa 702
NC_030191 Cryptocercus kyebangensis 703
NC_034841 Periplaneta australasiae 704
NC_029224 Blaptica dubia 705
NC_048888 Ostrinia zealis 706
NC_050874 Tuta absoluta 707
NC_041112 Clania variegata 708
NC_048887 Ostrinia scapulalis 709
NC_047456 Pontia edusa 710
KY_630500 Megathymus yuccae yuccae 711
MT_547768 Linoclostis gonatias 712
NC_012739 Antheraea yamamai 713
NC_018133 Actias selene 714

NC_022676 Spodoptera litura 715
NC_024532 Danaus chrysippus 716
NC_026903 Thitarodes gonggaensis 717
NC_027961 Plodia interpunctella 718
NC_030370 Helicoverpa zea 719
NC_034664 Junonia vestina 720
NC_038157 Vanessa indica 721
NC_045191 Pontia callidice 722
NC_045192 Pontia daplidice 723
NC_035890 Helicoverpa assulta 724
NC_039716 Ephestia elutella 725
NC_030632 Biston thibetaria 726
KX_087239 Anthrenus verbasci 727
NC_028207 Junonia coenia 728
NC_027071 Antheraea frithi 729
NC_024582 Grapholita dimorpha 730
NC_022685 Eurema hecabe 731
NC_019622 Spodoptera exigua 732
NC_046720 Clanis bilineata 733
KX_423729 Junonia genoveva genoveva 734
KY_628213 Cnidocampa flavescens 735
NC_044770 Thitarodes damxungensis 736
NC_046033 Gynanisa maja 737
NC_030270 Antheraea assama 738
NC_026532 Pieris canidia 739
NC_037874 Papilio slateri 740
NC_034663 Junonia litoralis 741
NC_027836 Spodoptera frugiperda 742
NC_010568 Pieris melete 743
NC_024407 Junonia almana 744
MN_623383 Junonia stygia 745
NC_018094 Thitarodes renzhiensis 746
NC_014055 Papilio maraho 747
MH_574940 Ostrinia palustralis 748
NC_010266 Manduca sexta 749
NC_037710 Junonia rhadama 750
NC_042150 Cydalima perspectalis 751
NC_018047 Papilio machaon 752
NC_047303 Pyralis farinalis 753
NC_022185 Agrotis ipsilon 754
NC_027723 Operophtera brumata 755
NC_026518 Bombyx huttoni 756

NC_023978 Papilio syfanius 757
NC_037725 Theretra japonica 758
NC_029716 Chilo sacchariphagus 759
NC_034356 Papilio rex 760
NC_043911 Papilio memnon 761
NC_033861 Junonia iphita 762
NC_034317 Papilio protenor 763
NC_034355 Papilio dardanus 764
NC_029193 Lobesia botrana 765
NC_027506 Papilio demoleus 766
NC_021416 Amata formosae 767
NC_021452 Danaus plexippus 768
NC_025763 Dendrolimus spectabilis 769
NC_023791 Helicoverpa punctigera 770
NC_028539 Heliothis subflexa 771
NC_016067 Phalera flavescens 772
NC_004622 Antheraea pernyi 773
MG_437199 Helicoverpa gelotopoeon 774
KX_267573 Junonia evarete zonalis 775
KX_267571 Junonia genoveva hilaris 776
MF_491642 Zeuzera multistrigata 777
NC_003395 Bombyx mandarina 778
NC_015895 Pieris rapae 779
NC_023530 Thitarodes pui 780
NC_025757 Papilio helenus 781
NC_037441 Macroglossum stellatarum 782
NC_037445 Psilogramma increta 783
NC_027252 Papilio glaucus 784
NC_041123 Sitotroga cerealella 785
NC_041106 Hydrillodes lentalis 786
NC_021411 Papilio maackii 787
NC_027157 Dendrolimus tabulaeformis 788
NC_028532 Galleria mellonella 789
NC_022697 Junonia orithya 790
NC_025551 Hamadryas epinome 791
MT_712074 Anartia jatrophae saturata 792
KY_630505 Megathymus beulahae beulahae 793
NC_015612 Chilo suppressalis 794
NC_003368 Ostrinia furnacalis 795
KT_876879 Agriotes obscurus 796
MF_346379 Saturnia jonasii 797
KX_267569 Junonia evarete flirtea 798

NC_037149 Bombyx lemeepauli 799
NC_003367 Ostrinia nubilalis 800
NC_014806 Grapholita molesta 801
NC_022689 Agrotis segetum 802
NC_020004 Biston panterinaria 803
NC_024742 Papilio polytes 804
NC_027156 Dendrolimus punctatus 805
NC_032285 Eurema blanda 806
NC_028348 Hepialus xiaojinensis 807
KY_630501 Megathymus streckeri streckeri 808
KF_927042 Actias artemis 809
KM_225795 Pectinophora gossypiella 810
KY_630502 Megathymus ursus violae 811
NC_002355 Bombyx mori 812
NC_014668 Helicoverpa armigera 813
NC_020003 Cydia pomonella 814
NC_022687 Catopsilia pomona 815
NC_024644 Chilo auricilius 816
NC_027111 Biston suppressaria 817
NC_028324 Junonia lemonias 818
NC_030769 Biston perclara 819
NC_036347 Dendrolimus kikuchii 820
NC_039840 Dendrolimus houi 821
NC_039841 Dendrolimus superans 822
NC_045899 Actias luna 823
NC_046032 Gonimbrasia belina 824
NC_041142 Pomacea 825
NC_027503 Pomacea maculata 826
NC_024586 Pomacea canaliculata 827
NC_037691 Pomacea bridgesii 828
NC_029207 Heterometrus longimanus 829
NC_008063 Nephila clavata 830
NC_025634 Araneus ventricosus 831
NC_032402 Araneus angulatus 832
NC_034027 Odontotermes obesus 833
NC_034108 Nasutitermes arborum 834
NC_034045 Nasutitermes octopilis 835
NC_034061 Odontotermes minutus 836
NC_030019 Coptotermes sepangensis 837
NC_030018 Coptotermes remotus 838
NC_034033 Cubitermes fulvus 839
NC_034035 Odontotermes mathuri 840

NC_034021 Microcerotermes newmani 841
NC_034065 Microcerotermes baluchistanicus 842
NC_034106 Odontotermes javanicus 843
NC_025522 Macrotermes natalensis 844
NC_009500 Reticulitermes virginicus 845
NC_030012 Coptotermes elisae 846
NC_030020 Coptotermes sjoestedti 847
NC_034133 Microcerotermes progrediens 848
NC_034109 Cubitermes sulcifrons 849
NC_034146 Nasutitermes macrocephalus 850
NC_009501 Reticulitermes hageni 851
NC_030013 Coptotermes frenchi 852
NC_034078 Macrotermes annandalei 853
NC_025567 Reticulitermes chinensis 854
KU_925198 Coptotermes acinaciformis acinaciformis 855
NC_026113 Cubitermes ugandensis 856
NC_015800 Coptotermes formosanus 857
NC_030014 Cotptotermes gestroi 858
NC_034055 Cornitermes pugnax 859
NC_030035 Reticulitermes grassei 860
NC_034115 Nasutitermes exitiosus 861
NC_034288 Macrotermes yunnanensis 862
NC_045231 Reticulitermes tibialis 863
NC_034121 Termes comis 864
NC_034142 Microcerotermes serrula 865
NC_034086 Cornitermes cumulans 866
NC_034042 Nasutitermes lujae 867
NC_034028 Odontotermes hainanensis 868
NC_034034 Nasutitermes matangensis 869
NC_034023 Nasutitermes longirostris 870
NC_031162 Reticulitermes flaviceps 871
NC_034093 Nasutitermes similis 872
NC_034060 Nasutitermes longipennis 873
NC_018599 Macrotermes barneyi 874
KU_925197 Coptotermes acinaciformis raffrayi 875
NC_034126 Protermes prorepens 876
NC_030017 Coptotermes michaelseni 877
NC_026115 Nasutitermes corniger 878
NC_034080 Nasutitermes neoparvus 879
NC_034024 Pseudacanthotermes spiniger 880
NC_030011 Coptotermes amanii 881
NC_028722 Coptotermes testaceus 882

NC_034046 Macrotermes carbonarius 883
NC_034056 Cubitermes oblectatus 884
NC_034043 Termes rostratus 885
NC_030021 Coptotermes travians 886
NC_034030 Macrotermes malaccensis 887
NC_034067 Microcerotermes fuscotibialis 888
NC_034020 Nasutitermes diabolus 889
NC_034110 Macrotermes gilvus 890
NC_034036 Microcerotermes crassus 891
NC_045240 Reticulitermes lucifugus 892
KY_484910 Reticulitermes speratus 893
NC_034050 Macrotermes falciger 894
NC_018125 Coptotermes lacteus 895
NC_034049 Termes fatalis 896
NC_018128 Macrotermes subhyalinus 897
MF_063063 Reticulitermes kanmonensis 898
NC_018131 Nasutitermes triodiae 899
NC_018122 Microhodotermes viator 900
NC_034127 Macrotermes muelleri 901
NC_037018 Coptotermes suzhouensis 902
NC_034104 Microcerotermes nervosus 903
NC_026114 Microcerotermes parvus 904
NC_034929 Labiotermes labralis 905
NC_009499 Reticulitermes santonensis 906
NC_026117 Termes hospes 907
NC_026695 Reticulitermes aculabialis 908
NC_009498 Reticulitermes flavipes 909
NC_030015 Coptotermes heimi 910
NC_034026 Nasutitermes banksi 911
NC_034054 Macrotermes vitrialatus 912
NC_034077 Pseudacanthotermes militaris 913
NC_034040 Nasutitermes graveolus 914
NC_034130 Odontotermes longignathus 915
NC_030036 Reticulitermes nelsonae 916
NC_042419 Reticulitermes leptomandibularis 917
NC_030016 Coptotermes kalshoveni 918
NC_042811 Mictis tenebrosa 919
NC_041655 Magicicada septendecula 920
NC_024581 Aphis gossypii 921
NC_028084 Nepa hoffmanni 922
NC_020373 Dolycoris baccarum 923
NC_039988 Aphis fabae mordvilkoi 924

MT_898422 Hyalopterus pruni
MN_316642 Aphis spiraecola
NC_041653 Magicicada cassini
NC_027194 Lethocerus indicus
NC_038148 Cacopsylla pyri
NC_011755 Nezara viridula
NC_041651 Magicicada neotredecim
NC_041652 Magicicada tredecim
NC_027087 Cacopsylla coccinea
NC_045886 Notonecta montandoni
NC_045236 Aphis glycines
NC_042441 Antilochus coquebertii
NC_037742 Tessaratoma papillosa
NC_037739 Cyclopelta parva
NC_026699 Darthula hardwickii
MG_488215 Cosmoscarta heros
NC_013272 Halyomorpha halys
KU_237288 Lethocerus deyrollei
KR_819174 Bemisia afer
MN_397939 Aphis aurantii
NC_042440 Antilochus russus
NC_041654 Magicicada tredecassini
NC_012817 Laccotrephes robustus
NC_042804 Catacanthus incarnatus
NC_042803 Cantao ocellatus
NC_006279 Bemisia tabaci
NC_031387 Aphis craccivora
MT_990978 Cacopsylla citrisuga
NC_040114 Cosmoscarta bispecularis
NC_042809 Leptoglossus membranaceus
NC_022449 Eusthenes cupreus
NC_041656 Magicicada tredecula
KY_817245 Scaphoideus varius
NC_042202 Erthesina fullo
NC_043903 Aphis citricidus
NC_040115 Cosmoscarta dorsimacula
KY_817244 Scaphoideus nigrivalveus
NC_035509 Anoplocnemis curvipes
NC_006280 Trialeurodes vaporariorum
NC_036671 Notonecta chinensis
NC_025495 Aeneolamia contigua
KY_817243 Scaphoideus titanus

NC_041650 Magicicada septendecim 967
NC_002084 Anopheles gambiae 968
NC_000875 Anopheles quadrimaculatus A 969
NC_014275 Anopheles darlingi 970
NC_006817 Aedes albopictus 971
NC_038158 Anopheles funestus 972
NC_036263 Anopheles dirus 973
NC_035159 Aedes aegypti 974
NC_028223 Anopheles stephensi 975
NC_015079 Culex pipiens pipiens 976
NC_014574 Culex quinquefasciatus 977
NC_022476 Ephestia kuehniella 978
NC_036678 Stegobium paniceum 979
NC_053358 Callosobruchus maculatus 980
KY_942061 Callosobruchus maculatus 981
NC_053876 Dermestes lardarius 982
NC_042820 Rhyzopertha dominica 983
MN_535903 Oryzaephilus surinamensis 984
JN_097761 Ptinus tectus 985
NC_026702 Tribolium confusum 986
NC_053875 Trogoderma granarium 987
KC_524798 Necrobia rufipes 988
NC_038197 Lasioderma serricorne 989
NC_030592 Abax parallelepipedus 990
NC_038132 Aacanthocnema dobsoni 991
NC_010532 Truginiophthalmus alternatus 992
NC_011717 Pedetontus silvestrii 993
NC_007688 Petrobius brevistylis 994
NC_051491 Pedetontus zhejiangensis 995
NC_006895 Nesomachilis australica 996
NC_021384 Songmachilis xinxiangensis 997
KJ_754501 Allophontus sp. 998
KJ_754502 Pedetontinus luanchuanensis 999
NC_046478 Ctenolepisma villosa 1000
NC_053634 Neoasterolepisma foreli 1001
NC_005437 Tricholepidion gertschi 1002
NC_047445 Lepisma saccharina 1003
NC_011197 Atelura formicaria 1004
NC_006080 Thermobia domestica 1005
AB_639034 Aposthonia japonica 1006
KX_091852 Eosembia sp. 1007
KX_091848 Aposthonia borneensis 1008

NC_018538 Challia fletcheri
KX_091853 Eudohrnia metallica
NC_032075 Euborellia arcanum
KX_091861 Paratimomenus flavocapitatus
KJ_778884 Doru luteipes
MG_253269 Euborellia annulipes
KX_673197 Anisolabididae sp.
NC_026077 Zorotypus medoensis
NC_036360 Dorypteryx domestica
NC_004816 Lepidopsocidae sp.
NC_036364 Lachesilla anna
NC_036365 Speleketor irwini
NC_036361 Prionoglaris stygia
NC_021400 Psococerastis albimaculata
NC_036362 Amphigerontia montivaga
NC_036363 Archipsocus nomas
NC_036366 Stimulopalpus japonicus
NC_023839 Liposcelis decolor
NC_015998 Anaticola crassicornis
NC_039530 Colpocephalum griffoneae
NC_015999 Ibidoecus bisignatus
NC_056779 Campanulotes compar
NC_025504 Liposcelis entomophila
NC_030594 Liposcelis keleri
NC_053761 Gynaikothrips ficorum
NC_050743 Pseudodendrothrips mori
NC_058008 Thrips hawaiiensis
NC_025241 Scirtothrips dorsalis
NC_035510 Anaphothrips obscurus
NC_018370 Frankliniella occidentalis
NC_037839 Dendrothrips minowai
NC_027488 Haplothrips aculeatus
NC_024657 Dysmicohermes ingens
NC_023444 Neochauliodes bowringi
NC_033349 Chloronia mirifica
NC_035504 Archichauliodes deceptor
NC_027852 Neoneuromus tonkinensis
NC_025281 Neochauliodes rotundatus
NC_025282 Neochauliodes fraternus
NC_027851 Nevromus exterior
NC_018772 Neochauliodes punctatolosus
NC_013256 Sialis hamata

NC_048517 Hemerobius japonicus
NC_028153 Neuronema laminatum
NC_019618 Chrysopa pallens
NC_030341 Chrysoperla externa
NC_013257 Ditaxis biseriata
NC_051001 Hemerobius spodipennis
NC_050653 Osmylus fulvicephalus
NC_053271 Gryposmylus pennyi
NC_021415 Thyridosmylus langii
NC_015095 Apochrysa matsumurae
NC_047285 Myrmeleon formicarius
NC_042677 Mircomus angulatus
NC_039771 Mantispa japonica
NC_015093 Chrysoperla nipponensis
NC_011278 Polystoechotes punctata
NC_023363 Rapisma zayuanum
NC_023362 Rapisma xizangense
NC_032298 Bullanga florida
NC_039948 Suhpalacsa longialata
NC_039773 Euclimacia badia
NC_015609 Libelloides macaronius
NC_011277 Ascaloptynx appendiculata
NC_021428 Ascalohybris subjacens
NC_025905 Epacanthaclisis banksi
NC_024826 Myrmeleon immanis
NC_039772 Eumantispa harmandi
NC_024825 Nymphes myrmeleonoides
NC_057219 Conwentzia sinica
NC_018545 Mengenilla moldrzyki
NC_040301 Ceratophyllus wui
NC_042380 Hystrichopsylla weida qinlingensis
NC_022710 Jellisonia amadoi
NC_036066 Dorcadia ioffi
NC_049858 Ctenocephalides felis
NC_025329 Hemiodoecus leai
NC_020309 Hackeriella veitchi
NC_021748 Nilaparvata lugens
NC_052691 Chloriona tateyamana
NC_056127 Sogatella kolophon
NC_021417 Sogatella furcifera
NC_042180 Sogatella vibix
NC_013706 Laodelphax striatellus

NC_037181 Changeondelphax velitchkovskyi
NC_052694 Perkinsiella saccharicida
NC_037182 Peregrinus maidis
NC_057613 Pyrops candelaria
NC_045075 Aphaena amabilis
NC_052695 Tropidocephala brunnipennis
NC_052693 Ishiharodelphax matsuyamensis
NC_042179 Saccharosydne procerus
NC_056249 Lycorma meliae
NC_012617 Geisha distinctissima
NC_051496 Ricania shantungensis
NC_031369 Ricania speculum
NC_052690 Bambusiphaga taibaishana
NC_052689 Bambusiphaga furca
NC_053739 Lophops carinata
NC_052692 Epeurysa nawaii
NC_012835 Lycorma delicatula
NC_033388 Nilaparvata bakeri
NC_024627 Nilaparvata muiri
